# Serum Concentrations of Cartilage Intermediate Layer Protein 2 Were Higher in Overweight and Obese Subjects

**DOI:** 10.1155/2022/6290064

**Published:** 2022-06-16

**Authors:** Qinge Li, Danlan Pu, Xuyun Xia, Hua Liu, Ling Li

**Affiliations:** ^1^The Key Laboratory of Laboratory Medical Diagnostics in the Ministry of Education and Department of Clinical Biochemistry, College of Laboratory Medicine, Chongqing Medical University, Chongqing, China; ^2^The People's Hospital of Yubei District of Chongqing City, China; ^3^Department of Pediatrics, School of Medicine, University of Mississippi Medical Center, USA

## Abstract

**Background:**

Cartilage intermediate layer protein 2 (CILP2) is associated with a variety of plasma lipoproteins and lipid traits. However, the correlation between CILP2 and obesity remains unknown. The aim of this study was to investigate the relationship between circulating CILP2 levels and obesity based on body mass index (BMI).

**Methods:**

A total of 252 subjects were divided into three groups: normal weight (*n* = 124), overweight (*n* = 94), and obese (*n* = 34). Metabolic parameters were measured in a fasting state. Serum CILP2 concentration was tested by enzyme-linked immunosorbent assay. Multivariate linear regression analysis was used to explore the relationship between CILP2 and obesity. We also conducted bioinformatics analysis to further explore the genes and signaling pathways related to CILP2.

**Results:**

The concentrations of serum CILP2 in the overweight and obese groups were significantly higher than that in the normal weight group. In multiple linear regression analysis, BMI was positively correlated with CILP2 concentration after controlling gender and age. Being overweight and obese were independently correlated with CILP2 concentration after adjusting for gender, age, SBP, DBP, FBG, 2-hour OGTT blood glucose (2h-BG), fasting blood insulin (FIns), TG, TC, HDL-C, LDL-C, and FFA. Bioinformatics analysis showed that the genes related to CILP2 are primarily associated with lipid metabolism and insulin resistance.

**Conclusion:**

We speculate that CILP2 may attribute to metabolic disorders in obesity.

## 1. Introduction

Obesity has become a worldwide epidemic, and it is considered to be an independent risk factor for many diseases, such as metabolic syndrome, diabetes, and cardiovascular disease, and even promotes tumorigenesis through fat inflammation, changes in the local microenvironment, and related changes in circulatory metabolism [1, 2]. The World Obesity Federation considered obesity as a chronic relapsing disease process [3]. Although it is controversial whether obesity should be recognized a disease, many authorities advocate that obesity is a noncommunicable disease [3]. In clinical practice, body mass index (BMI) is used to diagnose overweight and obesity [4]. Obesity is characterized by excessive accumulation of adipose tissue. More energy intake than energy consumption results in energy imbalance, which is an important reason for obesity [5].

Cartilage intermediate layer protein (CILP-1) was first identified in cartilage tissue. CILP-1 is synthesized by articular chondrocytes in explant culture. It is considered to be the product of chondrocytes. The distribution of CILP-1 in articular cartilage provides important information about the properties of different chondrocytes in related tissues, and its expression in articular cartilage increases with age [6]. CILP-1 is abundantly expressed in intervertebral discs, and it increases with the degeneration of the discs [7, 8]. CILP-1 modulates the susceptibility of lumbar disc diseases by regulating TGF-b signal transduction [7]. CILP2 is highly homologous to CILP-1, and their N-terminal domain contains a highly conserved thrombospondin type I repeat domain and immunoglobulin type C-2 domain [9]. Both CILP-1 and CILP2 are located in the extracellular matrix (ECM). CILP2 seems to be more abundant in the deeper middle region of the articular cartilage at maturity. CILP2 and CLIP-1 are also expressed in the skeletal muscle and heart [10].

Majority of the studies on CILP-1 focus on the regulation of musculoskeletal diseases and cardiac fibrosis [11–15]. A number of studies have shown that CILP2 is closely related to blood lipids. Earlier studies showed that the intergenic region between CILP2 and PBX4 is a new site for low-density lipoprotein cholesterol and triglyceride, and its single-nucleotide polymorphisms (SNPs) are related to a variety of lipoproteins and lipid traits [16]. Subsequent studies have confirmed that rs16996148 SNP located near CILP2 is associated with blood lipid levels [17–20]. We previously reported that CILP2 is a secretory protein and is highly expressed in patients with insulin resistance [21]. Since obesity is closely related to the disorders of glucose and lipid metabolism, we explored the relationship between human circulating CILP2 levels and overweight/obesity in this study.

## 2. Materials and Methods

### 2.1. Participants

In this cross-sectional study, a total of 252 subjects were randomly recruited from January 2017 to December 2017 by Hospital Management Information System (HIS). The participants were divided into three groups according to BMI, including normal weight (*n* = 124, 18.5 kg/m^2^ < BMI<24 kg/m^2^), overweight (*n* = 94, 24 kg/m^2^ ≤ BMI<28 kg/m^2^), and obesity (*n* = 34, BMI ≥28 kg/m^2^) [22]. There was no significant difference in age and gender composition among the three groups. Exclusion criteria included systemic inflammation or malignant tumor, history of diabetes and being treated with hypoglycemic agents, and receiving lipid-lowering drugs or antihypertensive drugs. All subjects provided written voluntary consent before participation. This study was approved by the ethics committee of Chongqing Medical University according to the declaration of Helsinki of the World Medical Association (Clinical Trial Registration No.: chictr-ocs-13003185).

### 2.2. Anthropometric, Biochemical, and CILP2 Measurements

Anthropometric measurements were performed in all participants before breakfast. Height, waist circumference, and hip circumference were measured to the nearest 0.1 cm and weight to the nearest 0.1 kg. BMI was calculated by dividing the height by the square of weight, and the waist-hip ratio is obtained by dividing waist circumference by hip circumference. The percentage of body fat (FAT%) was evaluated with bioelectrical impedance using the Tanita TBF-511 Body Fat Analyzer (Tanita Corporation, Tokyo, Japan). Blood glucose and glycated hemoglobin (HbA1c%) were tested by glucose oxidase method and anion-exchange high-performance liquid chromatography, respectively. Oral glucose tolerance tests (OGTT) were performed in all study subjects. At 7 a.m., all individuals restricted from food overnight were given glucose (75 g). Blood samples were taken at the designated time (0, 30, 60, and 120 min) for measuring blood glucose, including 2-hour OGTT blood glucose (2h-BG), insulin, and CILP2. Serum insulin was determined by radioimmunoassay. Free fatty acids (FFA) were measured using a commercial assay kit (Randox Laboratories Limited, Antrim, UK). The concentration of CILP2 in serum was detected by ELISA Kit (ELISA Biotech Co., Ltd). The detection limit was 6.25 ng/L, and the differences within and between tests were <8% and <10%, respectively. The linear range is 25~1600 ng/L. Verified by the manufacturer, the kit has high sensitivity and excellent specificity for the detection of human CILP2 without obvious cross-reaction or interference. Serum total cholesterol (TC), high-density lipoprotein cholesterol (HDL-C), low-density lipoprotein cholesterol (LDL-C), and triglyceride (TG) levels were measured by an automatic analyzer (Hitachi 747; Tokyo, Japan). The calculation formula of insulin resistance steady-state model is HOMA − IR = fasting insulin(*μ*U/mL) × Fasting blood glucose (mmol/L)/22.5.

### 2.3. Bioinformatics Analysis

Microarray data sets GSE24883, GSE20931, and GSE59034 in the GEO database were collected and analyzed for CILP2 mRNA expression in adipose tissue from obese and exercise or surgical weight loss subjects. The protein-protein interaction (PPI) network of CILP2 was constructed by using the STRING online database, and the lowest interaction score>0.4 was used for screening. David's online tool was used to enrich and analyze the gene ontology and signal pathway of associated proteins, and *p* < 0.05 was used to screen the analysis results of GO and KEGG terms.

### 2.4. Statistical Analysis

IBM SPSS Statistics 26 was used for statistical analysis. The continuous variables of the normal distribution are described by mean ± standard deviation (SD), and the continuous variables of skew distribution are expressed by median (interquartile interval). The classified variables are expressed as a percentage. A one-way analysis of variance (ANOVA), Kruskal Wallis, and Mann–Whitney *U* tests were used to analyze the differences among the groups. The chi-square test was used to analyze the differences in categorical variables among the groups. Multivariate linear regression analysis was used to determine the independent variables related to serum CILP2 concentration. We considered *p* value <0.05 is statistically significant.

## 3. Results

**Figure 1:**
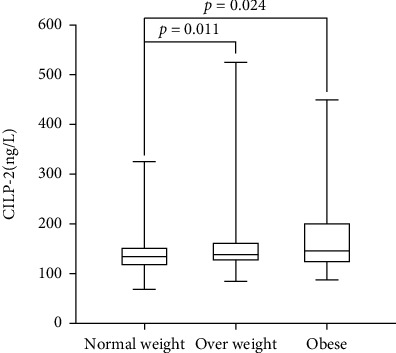
Comparison of serum CILP2 concentrations in normal weight, overweight, and obese subjects. Box plots of serum CILP2 concentrations in normal weight (*n* = 124), overweight (*n* = 94), and obese (*n* = 34). The lines in the box represent the median of the distribution, the values at the top and bottom of the box are defined by the 25th and 75th percentiles, and the lower and upper edges are the minimum and maximum values.

The average age of 252 subjects was 53.6 ± 10.4 years. Their baseline clinical characteristics are compared in Table 1. There were significant differences in body weight (*p* < 0.001), waist circumference (*p* < 0.001), BMI (*p* < 0.001), FAT (%) (*p* < 0.001), SBP (*p* < 0.001), DBP (*p* = 0.014), FBG (*p* = 0.041), fasting blood insulin (FIns) (*p* < 0.001), TG (*p* = 0.001), HDL-C (*p* = 0.006), and HOMA-IR (*p* < 0.001) among the three groups. The concentrations of CILP2 in normal weight, overweight, and obese groups were 133.56 (116.53-150.85) ng/L, 138.17 (125.79-164.34) ng/L, and 145.87 (122.41-201.60) ng/L, respectively. Further analysis by Mann–Whitney *U* test showed that the concentrations of serum CILP2 were significantly higher in overweight group (*p* = 0.011) and obese group (*p* = 0.024) as compared with normal body weight group (Figure 1).

In multiple linear regression analysis (Table 2), after adjusting for gender and age (model 1), BMI (*β* = 0.217, 95%CI = 0.094 − 0.339, *p* = 0.001) was positively correlated with serum CILP2 concentration. In order to evaluate whether BMI status had an effect on serum CILP2 concentration, the normal weight group was used as a reference and compared with the overweight and obese groups. We found that being overweight (*β* = 0.157, 95%CI = 0.034 − 0.279, *p* = 0.012) and obese (*β* = 0.132, 95%CI = 0.002 − 0.265, *p* = 0.047) were independently positively associated with CILP2 concentration after adjusting for gender, age, SBP, DBP, FBG, 2-hour OGTT blood glucose (2h-BG), fasting blood insulin (FIns), TG, TC, HDL-C, LDL-C, and FFA.

**Figure 2:**
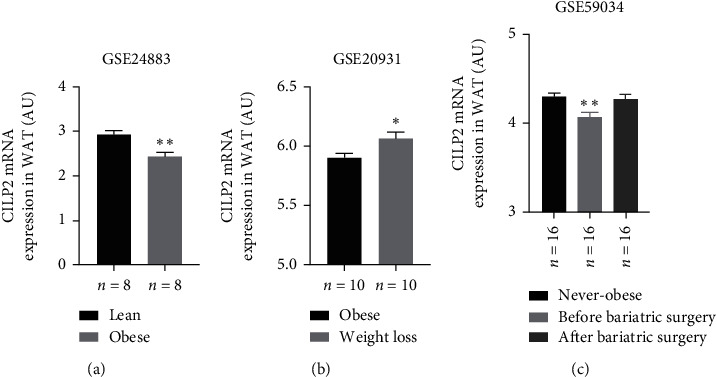
Evaluation of the expression level of CILP2 mRNA in human adipose tissue based on the open microarray data set. (a) GSE24883, expression of CILP2 in adipose tissue of obese and lean people. (b) CILP2 expression in adipose tissue of people who lose weight through exercise and succeed in obesity. (c) The expression of CILP2 in adipose tissue of nonobese and obese people, as well as obese people before and after weight loss surgery.

In order to further explore the relationship between CILP2 and obesity, we conducted an online bioinformatics analysis. First, we used a public microarray data set from the GEO database to evaluate the mRNA expression level of CILP2 in adipose tissue of obese and control groups. Interestingly, the mRNA level of CILP2 in adipose tissue of obese patients was significantly decreased as compared with the healthy control group (Figure 2(a)), and adipose CILP2 was significantly increased after weight loss resulting from exercise or weight loss surgery (Figures 2(b) and 2(c)). This is contrary to the higher level of circulating CILP2 in obese patients.

**Figure 3:**
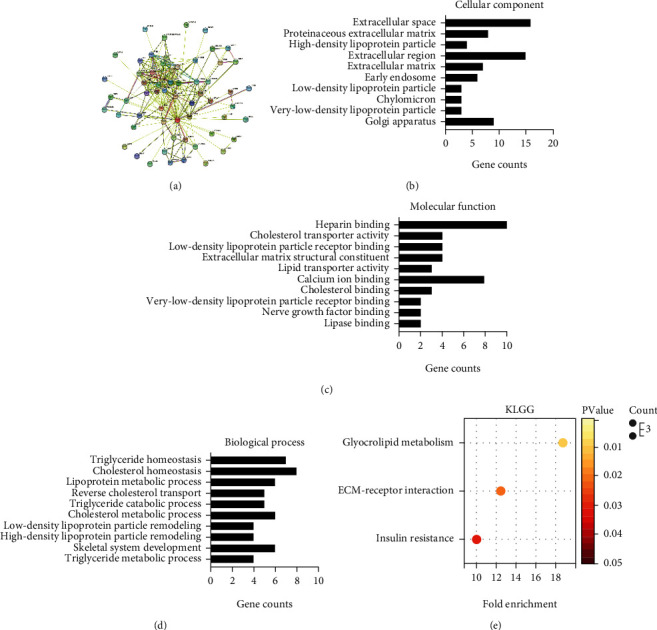
Bioinformatics analysis of CILP2 related proteins (genes) and signaling pathways. (a) Protein-protein interaction (PPI) network constructed by STRING database. Known interactions including experimentally determined and database obtained are pink edges and dark blue edges. The predicted interactions are green edge (gene neighborhood), blue edge (gene co-occurrence), and red edge (gene fusion). The yellow green edge is text mining. Black edges indicate common expression. Lavender edges indicate protein homology. (b) GO annotation analysis of cellular components (CC). (c) GO annotation analysis of molecular function (MF). (d) GO annotation analysis of biological process (BP). (e) KEGG pathway enrichment analysis. The gradient color represents the *p* value. The size of the bubble represents the number of genes.

Subsequently, we constructed a PPI network containing 59 proteins associated with CILP2 in the STRING database (Figure 3(a)). The 10 proteins closely associated with CILP2 were PBX4, TM6SF2, GTPBP3, TRIB1, FURIN, GALNT2, ANGPTL3, NCAN, PSRC1, and CELSR2. GO analysis showed that most of these genes were involved in the formation of extracellular regions, lipoprotein particles, early endosomes, and Golgi bodies (Figure 3(b)). The molecular functions of these proteins are mainly related to the binding function and the transport of lipoprotein and cholesterol (Figure 3(c)). Biological processes that the proteins involved include three aspects: (i) triglyceride and cholesterol homeostasis; (ii) metabolism of triglyceride, cholesterol, and lipoprotein; (iii) and skeletal muscle phylogeny (Figure 3(d)). KEGG signaling pathway analysis showed that these genes were mainly enriched in glycerol metabolism, ECM receptor interaction, and insulin resistance (Figure 3(e)).

## 4. Discussion

This article is the first study to explore the differences in CILP2 levels among normal weight, overweight, and obese people with BMI as the criterion. Compared with the normal weight group, the levels of serum CILP2 in overweight and obese groups were significantly higher, suggesting a potential association between CILP2 and obesity. Furthermore, after controlling gender and age, serum CILP2 level was positively correlated with BMI. In the public microarray data set of the GEO database, the mRNA of CILP2 in adipose tissue of obese patients is downregulated, which is contrary to the increasing trend of CILP2 in circulation, suggesting that serum CILP2 of obese patients comes from tissues other than adipose tissue. From the STRING databases, the proteins that have high interaction scores with CILP2, such as TM6SF2 and ANGPTL3, are closely related to lipid metabolism [23–26].

A study showed that there were differences in genotype and allele frequency of rs16996148 SNP located in NCAN/CILP2/PBX4 fragment between Mulao and Han populations in Guangxi province, China. There were also differences in the relationship between rs16996148 SNP and blood lipid level. The relationship between rs16996148 SNP of gender (male)-specific NCAN/CILP2/PBX4 and blood lipid level was found in both ethnic groups [17]. Another study in Southwest China demonstrated that the genotype and allele frequency of SNP locus located in CILP2 are significantly different between patients with hyperlipidemia and normal subjects. This gene locus has been shown to be able to change the risk of hyperlipidemia by interacting with other genes or with the environment [18]. Luptakova et al. described the profound effects of the CILP2 gene on HDL-C, LDL-C, apoB, non-HDL-C levels, and three atherosclerosis indicators in Slovak women, but no significant effect on triglyceride levels [19]. A missense single-nucleotide polymorphism rs58542926 between the NCAN-CILP2 region and TM6SF2 gene was observed in patients with nonalcoholic fatty liver in East Asian ethnic groups and Japanese, and this missense SNP was genetically attributed to the blood lipid level in the patients [20]. Even though more and more evidence showed that the CILP2 gene is closely related to blood lipid levels, how it affects blood lipid traits is still unknown.

It has been reported that CILP2 increased the lipids uptake in THP-1 cells and promoted the transformation of THP-1 cells into foam cells by positively regulating the transcription of CD36 via PPAR*γ*. This process contributes the formation of atherosclerosis [27]. CD36 is a clearance receptor that plays a role in the uptake of long-chain fatty acids (FAs) by high-affinity tissues and promotes lipid accumulation and metabolic disorders under excessive fat supply [28]. In adipocytes, intestinal cells, cardiomyocytes, and skeletal muscle cells, CD36 seemed to be the main membrane protein promoting fatty acid transport [29]. Further investigations are needed to explore whether CILP2 can affect lipid metabolism through CD36 in adipose tissue, skeletal muscle, and other tissues under obesity.

In our previous study, we found that CILP2 was associated with insulin resistance. CILP2 affected hepatic gluconeogenesis and insulin sensitivity by positively regulating the transcription of PEPCK [21]. Interestingly, this study also demonstrated that CILP2 mRNA and protein levels were downregulated in adipose tissue of diet-induced obese mice [21]. This result is consistent with the lower level of CILP2 mRNA in adipose tissue of obese patients from the public microarray data set from the GEO database, while it is opposite to the higher level of serum CILP2 in obese subjects. We speculate that the decreased expression of CILP2 in adipose tissue is a compensatory effect in response to the occurrence of obesity [30]. Since the CILP2 level in adipose tissue was not measured in the current study, the relationship between serum CILP2 level and adipose tissue CILP2 level could not be directly examined. More detailed research is needed to explore the effect of tissue CILP2 level on obesity. In addition, our study is a cross-sectional study without considering the causal relationship between serum CILP2 levels and obesity.

## 5. Conclusion

Our study showed that the level of serum CILP2 was positively correlated with BMI. The concentration of serum CILP2 in overweight and obese subjects was higher than that in normal subjects. Bioinformatics analysis also showed that CILP2 was closely related to lipid metabolism. Further studies are needed to explore the pathophysiological role of CILP2 in obesity and its effect on glucose and lipid metabolism.

## Figures and Tables

**Table 1:** Comparison of clinical parameters in normal weight, overweight and obese subjects.

Clinical parameters	Normal weight (*n* = 124)	Overweight (*n* = 94)	Obese (*n* = 34)	*p*
Age (years)	54.2 ± 10.3	53.1 ± 10.2	52.9 ± 11.6	0.679
Male (%)	33.9	42.5	35.3	0.408
Diabetes (%)	31.4	36.2	41.2	0.523
Body weight (kg)	57.0 ± 6.7	66.6 ± 7.4	75.0 ± 8.9	<0.001
Waist circumference (cm)	82.19 ± 8.12	90.10 ± 7.20	94.54 ± 6.84	<0.001
WHR	0.87 ± 0.06	0.91 ± 0.05	0.90 ± 0.04	<0.001
BMI (kg/m^2^)	21.87 ± 1.40	25.61 ± 1.13	29.97 ± 2.19	<0.001
FAT (%)	28.65 (23.60-32.10)	34.45 (25.70-37.00)	41.05 (31.28-42.65)	<0.001
SBP (mmHg)	121.6 ± 16.3	122.2 ± 15.3	134.3 ± 18.4	<0.001
DBP (mmHg)	75.5 ± 10.3	76.9 ± 9.4	81.2 ± 8.9	0.014
FBG (mmol/L)	5.53 (5.00-6.36)	5.78 (5.28-7.12)	6.41 (5.21-7.34)	0.041
2h-BG (mmol/L)	8.65 (6.90-12.20)	9.38 (7.10-12.38)	9.92 (7.54-13.25)	0.351
FIns (mu/L)	6.58 (5.12-9.22)	8.80 (5.88-12.43)	12.73 (8.82-19.41)	<0.001
HbA1c (%)	5.90 (5.50-6.40)	6.10 (5.70-6.80)	6.30 (5.70-6.95)	0.086
TG (mmol/L)	1.22 (0.88-1.80)	1.47 (1.08-2.16)	1.80 (1.19-2.66)	0.001
TC (mmol/L)	5.00 ± 1.19	5.08 ± 0.95	5.22 ± 1.07	0.556
HDL-C (mmol/L)	1.36 ± 0.33	1.23 ± 0.24	1.35 ± 0.47	0.006
LDL-C (mmol/L)	3.07 ± 1.08	2.97 ± 0.76	3.24 ± 0.79	0.339
FFA (*μ*mol/L)	0.57 ± 0.28	0.57 ± 0.24	0.59 ± 0.20	0.933
HOMA-IR	1.74 (1.25-2.78)	2.39 (1.50-4.01)	3.88 (2.54-5.61)	<0.001

Values are given as mean ± SE or median (interquartile range). WHR: waist-to-hip ratio; BMI: body mass index; SBP: systolic blood pressure; DBP: diastolic blood pressure; FBG: fasting blood glucose; 2h-BG: 2-hour OGTT blood glucose; FIns: fasting blood insulin; TG: triglyceride; TC: total cholesterol; LDL-C: low-density lipoprotein cholesterol; HDL-C: high-density lipoprotein cholesterol; FFA: free fatty acid; HOMA-IR: homeostasis model assessment of insulin resistance.

**Table 2:** Results of multivariate linear regression analysis between serum CILP2 and clinical variables.

	Model 1		Model 2	
	*β* (95% CI)	*p*	*β* (95% CI)	*p*
Age	-0.051 (-0.173~0.071)	0.411		
Sex	0.070 (0.052~0.192)	0.261		
BMI	0.217 (0.094~0.339)	0.001		
OW vs. NW			0.157 (0.034~0.279)	0.012
OB vs. NW			0.132 (0.002~0.265)	0.047
SBP				
DBP				
FBG				
2h-BG			0.267 (0.153~0.380)	<0.001
FIns			0.227 (0.102~0.352)	<0.001
TG				
TC				
HDL-C				
LDL-C				
FFA				

OW: overweight; NW: normal weight; OB: obese.

## Data Availability

The original contributions presented in the study are included in the article, and further inquiries can be directed to the corresponding author.

## Abbreviations

BMI: Body mass index
FBG: Fasting blood glucose
2h-BG: 2-hour OGTT blood glucose
FIns: Fasting serum insulin
TG: Triglyceride
HDL-C: High-density lipoprotein cholesterol
FFA: Free fatty acid
HOMA-IR: Homeostasis model assessment of insulin resistance.
